# Dietary knowledge, attitude and practice among type 2 diabetes mellitus patients in Sudan: a hospital-based cross-sectional study

**DOI:** 10.4314/ahs.v21i1.6

**Published:** 2021-03

**Authors:** Halla Mahagoub Idrees Adam, Yousif Mohammed Elmosaad, Abd Elbasit Elawad Ahmed, Asif Khan, Ilias Mahmud

**Affiliations:** 1 Department of Health Education, Faculty of Public and Environmental Health, University of Khartoum, Khartoum, Sudan; 2 College of Applied Medical Sciences, King Faisal University, KSA; 3 Department of Public Health, College of Public Health and Health Informatics, Qassim University, Bukayriyah, Qassim, Saudia Arabia

**Keywords:** Dietary knowledge, type 2 diabetes mellitus, Sudan

## Abstract

**Background:**

In Sudan, the prevalence of diabetes in adults was estimated at 19.1% in 2015. This study assessed dietary knowledge, attitude, and practices (KAP) of type 2 diabetes mellitus (T2DM) patients in Sudan.

**Methods:**

We randomly selected 238 T2DM patients from a list of 2460 patients from the Jabber Abulizz Hospital. We interviewed them face-to-face using a structured questionnaire. Multivariate logistic regression analyses were performed to investigate the determinants of KAP regarding the recommended diets for T2DM patients.

**Results:**

Majority of the patients demonstrated good knowledge (54.6%), positive attitude (79%); and good practice (58%). The result revealed that patients with formal education had 3.0 (95% CI: 1.6–5.7) times higher odds of having good diabetic dietary knowledge than those with informal education. While patients who had good knowledge and a positive attitude were respectively 4.7 (95% CI: 2.4–8.9) and 3.2 (95% CI: 1.5–6.7) times more likely to follow dietary recommendations than the patients with poor knowledge and negative attitudes.

**Conclusion:**

Irrespective of the socio-demographic position, the good knowledge and the positive attitude towards the recommended diet, all the T2DM patients complied with the dietary recommendations. These findings highlight the need for improving knowledge and promoting positive attitudes towards the recommended diet among T2DM patients.

## Background

Globally, diabetes is one of the major chronic disease burdens. Approximately 415 million adults aged 20–79 years are living with diabetes 1. Around three-quarters of them live in low- and middle-income countries. Research from various countries predicts that the diabetes epidemic will continue to increase 2, 3. By the year 2040, the prevalence of diabetes is estimated to rise to 642 million globally. The highest rise in prevalence is estimated to take place in the regions graduating from low-income to middle-income levels 1. Changes in lifestyles due to demographic transition, rapid urbanization and industrialization have been the cause of increased diabetes prevalence in these countries 1, 4.

The Middle East has a very high (11%) prevalence of diabetes 5. Six of the top 10 countries with the highest prevalence of diabetes are in the Arab region namely Kuwait (21.1%), Lebanon (20.2%), Qatar (20.2%), Saudi Arabia (20%), Bahrain (19.9%) followed by United Arab Emirates (UAE) (19.2%) 5. In this region, nearly three-fourths (73.4%) of the people living with diabetes are aged under 60 years and are at the peak of their productive years (40 years of age). Thus, diabetes becomes a real burden that results in productivity loss. In contrast, most people with diabetes in developed countries are above the age of retirement, and hence there is less impact on their productivity 5.

A lack of physical activity, high carbohydrate intake, aging populations and social determinants which include education, housing, and access to nutritious food^6^ have been attributed to an increased prevalence of diabetes^7^. In 2019, the International Diabetes Federation's (IDF's) reported that 19.4 million adults in Africa had diabetes and this figure is expected to rise to about 28 million by 2030 and 41.5 million in 2035. The apparent increase in diabetes prevalence is related to economic development^8^,^9^. Some of the most populous countries in Africa have the highest number of people with diabetes, including South Africa (4.6 million), Nigeria (2.7 million), Democratic Republic of Congo (1.8 million) and Ethiopia (1.7 million) 8.

In Sudan, the prevalence of diabetes in adults was 7.7% in 2013 1 and rose to 19.1% in 2015 10. This more than two-fold rise of diabetes prevalence has been attributed to aging, obesity, a diet that is unhealthy, low physical activity and rapid urbanization^10^.

Diabetes is a silent disease; people often remain unaware of their diabetes status. The majority of diabetes patients become aware of their diabetes only after they develop one of its life-threatening complications 11. Appropriate nutritional knowledge, attitude and practices regarding diabetes mellitus (DM) can delay the onset, delay and control the progression of complications 12, 13. Evidence suggests that the most effective strategy for the management of diabetes and its complications is patient's education 14, 15. Vital and effective choices that affect the diabetic patients' health and well-being are not made by their health service providers, but by the patients themselves 16. Therefore, a sound understanding of dietary knowledge, attitudes and practices of people living with diabetes can be instrumental in the overall diabetes management. The educational strategies used to facilitate appropriate dietary practices among people living with diabetes will probably be more effective if we understand the patients' knowledge, attitudes and practices regarding food consumption. These will assist in policy development, implementation of programmes and techniques for effective health education for people living with diabetes.

In the current study, we aimed to assess the knowledge, attitudes and practices of diabetic patients about their diet and investigate the determinants of their KAP.

## Design and methods

This cross-sectional study was conducted on people with T2DM who attend Jabber Abulizz Hospital, Khartoum State, Sudan. This hospital was established in 1998. It is a governmental hospital specializing in the treatment of diabetes and is managed by the Ministry of Health. The average daily patients' attendance is 82 patients.

A total of 238 diabetic patients attending the Jabber Abulizz Hospital between August and September 2015 were recruited in the study. The Jabbir Abulizz Hospital was purposively selected, but the study participants, diabetic patients attending the hospital, were selected through systematic random sampling. We used a sampling frame of 2460 patients based on the number of average monthly attendance of diabetic patients in that hospital and a sampling fraction of 10, dividing the sampling frame by the estimated sample size of 238 patients to approach diabetic patients to participate in the study. We calculated the sample size using the formula n= z^2^pq/d^2^. Where: n = sample size; Z = corresponding value to the 95% confidence level (1.96); P = previous rate taken from previous studies (0.192) (17); q = 1-p =1–0.192= 0.808 and d = desired margin of error = 0.05 17.

### Data collection

A structured questionnaire was used in data collection. The questionnaire covered questions regarding dietary knowledge, attitudes and practices (KAP) of the Type 2 diabetes mellitus (T2DM) patients. In addition to KAP, we collected socio-demographic data that included information related to age, gender educational attainment, marital status, income and number of family members as factors that influence KAP and the dietary choices. The questionnaire consisted of 25 questions of which 15 questions were related to knowledge, one question was related to practice and four questions were related to attitudes about diet for T2DM patients. The data on each part of the questionnaire was obtained by face-to-face interview with the interviewer.

For knowledge, 15 was the maximum possible score in which each correct answer was given one point and an incorrect or an answer of which the patient was not sure, was given zero point. The maximum possible score for attitude was four where each positive attitude was given a solitary point while a negative attitude was not awarded any point. Regarding practice, we only asked whether the person was following the recommendations provided by the health service providers in that hospital regarding their diet. Based on the previous studies^18^, ^19^,^20^,^21^ and the Modified Bloom's cut off points 22 to classify knowledge as poor knowledge and give a negative attitude a score of less than the mean score while the mean or above mean scores were considered as good knowledge and a positive attitude.

The protocol for this cross-sectional study was approved by the Ethical Committee of the Medical and Health Studies Board of the University of Khartoum. Verbal informed consent was obtained from the participants. Privacy and anonymity were maintained at the highest level throughout the research and publication processes.

### Data analysis

Data were analyzed using the Statistical Package for Social Science (SPSS) version 20. All statistical tests were performed considering 0.05 as the level of significance. Descriptive analyses were performed to present the demographic characteristics of the participants. For descriptive analyses, both frequency and proportions were presented in tables. Multivariate logistic regression was performed to investigate the association between the sociodemographic variables included in the models such as age, sex, marital status, household monthly income and family size and knowledge, attitudes and practice related to diabetic diets. OR and its 95% CI were estimated for each variable. A p-value <0.05 (two-sided) was considered to be statistically significant.

## Results

Table 1 shows the socio-demographic characteristics of the respondents. A total of 238 T2DM patients consented and participated in the study. The majority of the participants (56.6%) were females, (79%) of them were older than 40 years; had at least some formal education (69.3%) and 2 to 6 members in their family (55.5%). The monthly household income of over a third of the patients (34.5%) was less than 37.6 USD.

**Table 1:** Socio-Demographic Characteristics-A Cross-Sectional Survey of T2DM Patients, Jabber Abulizz Hospital, Sudan, 2015

Socio-Demographic Characteristics	Count	Per cent
**Age Group**		
18 – 40 years	50	21
More than 40 years	188	79
**Gender**		
Male	101	42.4
Female	137	56.6
**Education Level**		
Informal education ¥	73	30.7
Formal education ƒ	165	69.3
**Household Monthly Income**		
Less than 250 Sudanese Pound (SP)*	82	34.5
250–500 SP	94	39.5
More than 500 SP	62	26.1
**Family Size**		
2 – 6 members	132	55.5
More than 6 members	106	44.5

* 1 USD= 6.65 Sudanese Pound

¥ Informal education; any non-certified education ((Khalwa and self-learning (read and write))

ƒ Formal education: includes: primary, secondary and university and above

Our multivariate analysis showed that the dietary knowledge of the diabetic patients has a statistically significant association with education, gender and household monthly income while no statistically significant association was observed between dietary knowledge, marital status, and family size. We observed that patients with at least some formal education had 3.0 (95% CI: 1.6–5.7) times higher odds of having good diabetic dietary knowledge than those with informal education while male patients had 2.5 (95% CI: 1.4–4.5) times higher odds of having good dietary knowledge than female patients. Furthermore, patients with a monthly household income of 250–500 Sudanese pounds and more than 500 Sudanese pounds respectively have 2.6 (95% CI: 1.4–5.1) and 2.3 (95% CI: 1.1–4.8) times higher odds of having good dietary knowledge than those with a monthly household income of less than 250 Sudanese pounds. However, a statistically significant association between the socio-demographic variables and attitudes of the T2DM patients towards recommended diets was not found. Further details of this analysis are presented in Table 2.

**Table 2:** Socio-demographic determinants of dietary knowledge and attitude-a cross-sectional survey of T2DM Patients, Jabber Abulizz Hospital, Sudan, 2015

	Knowledge	Attitude
**Variables**	**Odds Ratio (95% CI)**	**Odds Ratio (95% CI)**
**Age**		
Up to 40 years	1	1
More than 40 years	0.837 (0.404–1.736)	0.985 (0.433–2.239)
**Education**		
Informal	1	1
Formal*	3.037) 1.625–5.678(	1.671(0.840–3.323)
**Marital Status**		
Married	1	
Unmarried	1.176(0.582–2.379)	1.115(0.502–2.479)
**Sex**		
Female	1	1
Male*	2.505(1.395–4.498)	1.156 (0.603–2.216)
**Monthly Household Income**		
Less than 250 SP	1	1
250–500 SP*	2.629(1.351–5.115)	0.954(0.438–2.078)
More than 500 SP*	2.325(1.127–4.796)	0.738(0.326–1.671)
**Family Size**		
up to 6 members	1	1
More than 6 members	1.430(0.787–2.596)	1.407(0.711–2.785)

Our multivariate logistic regression analysis suggested a significant association between adherence to diet recommendations and knowledge, attitudse, age, and income of diabetic patients. We observed that those who had good knowledge and positive attitudes were respectively 4.7 (95% CI: 2.4–8.9) and 3.2 (95% CI: 1.5–6.7) times likely to adhere to dietary recommendations than the patients with poor knowledge and negative attitudes respectively, adjusting for socio-demographic variables. Patients who were older than 40 years were 2.4 (95% CI: 1.1–5.3) times likely to comply with the recommended diet than the younger group. In comparison with the patients with less than 250 SP, patients with a monthly income of 250–500 SP and over 500 SP were respectively 2.9 (95% CI: 1.4–5.9) and 2.5 (95% CI: 1.1–5.7) times likely to comply with the recommended diets.

## Discussion

Our study is a hospital-based cross-sectional study; conducted among T2DM patients attending the Jabbir Abulizz Hospital, a specialist government hospital for T2DM treatment and management in Khartoum, Sudan. It was conducted to ascertain the dietary knowledge, attitudes and practices of T2DM patients. We found that over half of the participating patients had good dietary knowledge; hence were aware of their recommended diet. Similarly, a hospital-based survey among diabetic patients in Nasambaya, Kampala found that 54% of the participants had good dietary knowledge^23^.

However, the proportion of people who had good knowledge regarding diabetic diet as reported by our study is higher than the proportion of people who had good overall knowledge regarding diabetes as reported by community-based studies conducted in Kenya (27.2%) 24 and Sudan (15.0%) 25. The differences are probably because we measured knowledge among DM patients while these studies measured knowledge in the community. Besides, our study just focused on dietary knowledge while the studies referred to focus on the overall diabetes knowledge. Our findings are consistent with that of another institution-based survey on diabetic-related health knowledge in Nepal which reported that about 44% of patients had insufficient knowledge^26^. While another institution-based survey among newly diagnosed T2DM patient reported that only 18% of the respondents had poor knowledge (< mean - 1 SD)^12^. Ours is a hospital-based KAP survey of T2DM patients regarding their diet; however, about half of the respondents did not demonstrate good dietary knowledge. This might indicate that there is a need to improve communication between patients and the health services providers. Improved knowledge can bring positive changes in the attitudes and practices thus leading to better management of the disease and better health outcomes 27. The lack of proper knowledge among diabetic patients regarding their diet should be given attention for better management of T2DM since we found that patients with good knowledge are 4.7 times more likely to adhere to the recommended diet.

As far as attitudes are concerned, our finding showed that the majority of the study participants reported positive attitude towards the recommended diet although just over half of the participants were complying with the recommended diet. Other studies conducted in Iran (47.1%) 28, KSA (41.5%)^29^ and Pakistan (26.8%) 30 reported that less than half of the participants had positive attitudes towards the recommended diet. Positive attitudes lead to good practice which is also supported by our study findings. Patients with positive attitudes demonstrated 3.2 times higher odds of following the recommended diet than those with negative attitudes after adjusting for knowledge and socio-demographic variables.

Our study found that more than fifty percent of the patients were following the recommended diet. This result is in agreement with studies conducted in Nigeria 31, Iran 32, and UAE 33. However, it is a matter of concern, since almost half of the patients were not adhering to the recommended diet which will have negative consequences on their health outcome.

We found that the dietary knowledge of diabetic patients had a statistically significant association with education, gender and household monthly income. KAP studies of diabetic patients conducted in Bangladesh and UAE also stated that a higher level of education in patients is associated with a higher level of knowledge regarding diabetes 16, 34. We found that males had better knowledge regarding diet than females. This could be because women often receive inferior healthcare services 34; hence maybe they do not receive appropriate counseling and information regarding their diet. Also, we found that patients with higher household income demonstrated better knowledge. Studies conducted in Northwest Ethiopia^35^ and in a different context, rural Bangladesh 34 had the same findings. Perhaps, this is because a higher income leads to a better approach and higher access to information on diabetes which in turn might result in a changed behavior among the patients^37^.

Though we found that knowledge was associated with some socio-demographic variables, we could not find any evidence of an association between T2DM patients' attitudes towards the recommended diet and socio-demographic variables. Many previous studies in different settings likBangladesh, UAE and Southern Sri Lanka have reported that the level of attitude towards diabetes was not significantly associated with the education level^16^, ^34^, ^38^. However, in a cross-sectional study like this, it is not evident to find the reasons for the absence of the association between attitude and socio-demographic variables because may be there is several factors, such as the community culture, could be a reason.

Nevertheless, other studies reported that the subjects' knowledge determined their attitudes which therefore affected their practices towards dietary intake 33. Health education messages provided to the patients by the health care providers perhaps play an important role in developing positive attitudes towards the recommended diets irrespective of their socio-demographic background.

Regarding dietary practice, we found that it is significantly associated with the patients' level of knowledge, attitude, age and household income. Patients with good knowledge and a positive attitude were 4.7 and 3.2 times more likely to follow dietary recommendations than the patients with poor knowledge and negative attitudes respectively. A KAP study conducted in the UAE found lower diabetes awareness along with positive attitudes about the importance of care in diabetes and acceptable diabetes practices. They found a significant association between knowledge, attitude and practice, but the correlation was weak 33. This study reported a very high proportion of positive attitudes towards diabetes care. Often these kinds of questions are subject to social desirability bias. Hence, they might find a weak correlation between knowledge, attitude and practice. Nevertheless, in our study, we found a strong association between knowledge and practice; and also between attitude and practice. This has a far-reaching implication for diabetes care. Health care providers should invest time in education and counselling patients regarding dietary requirements to facilitate good practices and positive health outcomes.

## Conclusion

Our study found that about half of the T2DM patients had poor knowledge, one-fifth had a negative attitude and over two-fifths were not following the recommended diet. There was evidence of an association between following the recommended diet and good knowledge and a positive attitude towards the recommended diet, after controlling the socio-demographic variables. Individuals with good dietary knowledge are 4.7 times more likely to follow the recommended diet compared to those with poor knowledge. In addition, individuals with positive attitudes towards the recommended diet are 3.2 times more likely to follow the diet recommended by the hospitals compared to those with negative attitudes. Hence, the study findings highlight the need for improved health education and counselling pertaining to the appropriate diets for T2DM patients in hospitals.

## Figures and Tables

**Table 3:** Determinants of Adherence to a Recommended Diet - a cross-sectional survey of T2DM Patients, Jabber Abulizz Hospital, 2015

Variables	OR	95% C.I. for OR	
		Lower	Upper
**Knowledge**			
Poor	1		
Good*	4.659	2.449	8.866
**Attitudes**			
Negative	1		
Positive*	3.181	1.514	6.684
**Age**			
Up to 40 years	1		
More than 40 years*	2.402	1.092	5.284
**Education**			
Informal	1		
Formal	1.221	.606	2.463
**Family Size**			
Up to 6 members	1		
More than 6 members	1.073	.554	2.078
**Marital Status**			
Married	1		
Unmarried	.749	.361	1.553
**Monthly Household Income**			
Less than 250 SP	1		
250–500 SP*	2.872	1.391	5.929
More than 500 SP*	2.497	1.103	5.651
**Sex**			
Female	1		
Male	1.519	.799	2.886

## List of abbreviations

CI: Confident Interval; KAP: Knowledge, Attitude and Practice; OR: Odds Ratio; S.E: Standard Error; SP: Sudanese Pound; SPSS: Statistical Package for Social Sciences; T2DM: Type 2 Diabetes Mellitus; UAE: United Arab Emirates; USD: United States Dollar

## Declarations

### Availability of data and material

Data is available on reasonable request from the corresponding author.
